# Supplementary material for: Hypoxic regulation of chromatin and gene transcription
Source: Commun Biol. 2026 Mar 25;9:665. doi: 10.1038/s42003-026-09875-6 (PMC13181004; doi:10.1038/s42003-026-09875-6)
Supplement: Supplementary file 1 — Supplementary Information [file 42003_2026_9875_MOESM1_ESM.pdf]

Supplementary Information

Supplementary figure 1

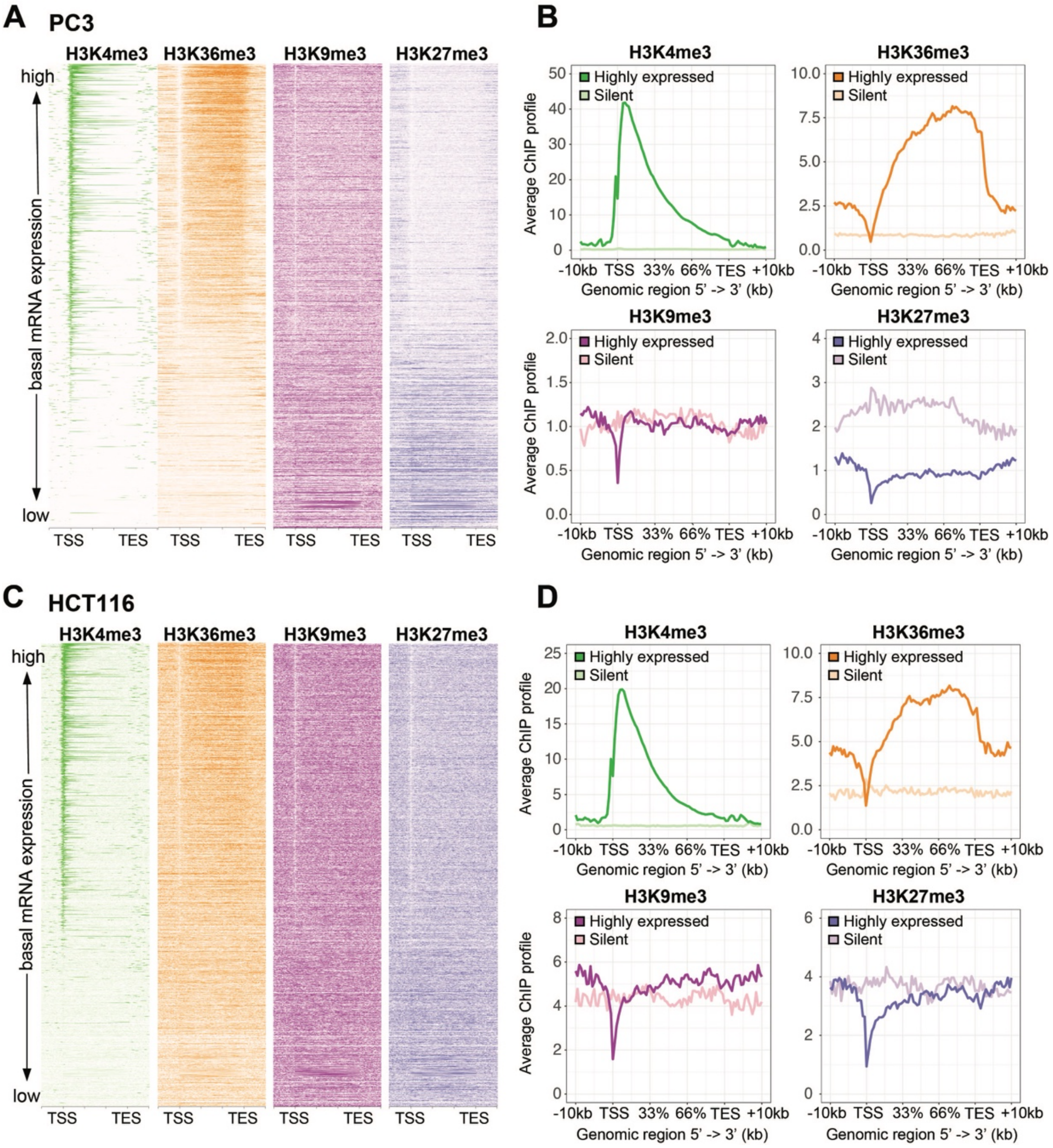

Supplementary figure 1. Basal levels of histone trimethylation (in normoxia) correlate with basal levels of expression. (A and C) Heatmaps and (B and D) average ChIP profiles displaying basal normoxic H3K4me3, H3K36me3, H3K9me3, H3K27me3, and total histone H3 ChIP-seq signal across all genes (±10kb). Each row denotes the signal across a single gene locus ranked from high basal expression (top) to low and silent (bottom) in (A and B) PC3 cells and (B and D) HCT116 cells.

Supplementary figure 2

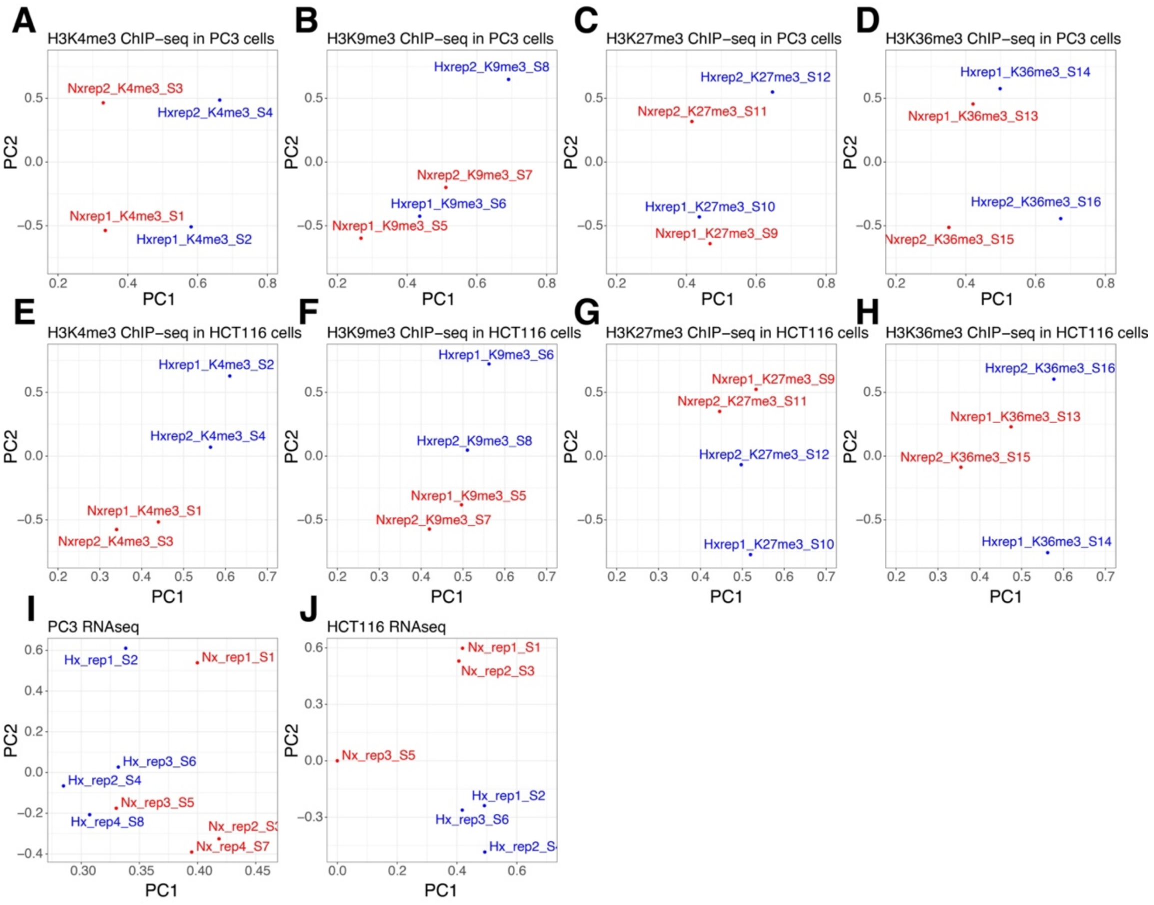

Supplementary figure 2. Principal component analysis (PCA) plots for intragenic histone methylation ChIP-seq and RNA-seq signals. ChIP-seq reads were mapped to individual gene loci and normalised to Drosphila spike in for each sample. PCA was performed for (A) H3K4me3, (B) H3K9me3, (C) H3K27me3 and (D) H3K36me3 signal in PC3 cells and (E-H) the same signal in HCT116 cells. (I & J) show PCA plots for RNA-seq analysis of PC3 and HCT116 cells respectively.

Supplementary figure 3

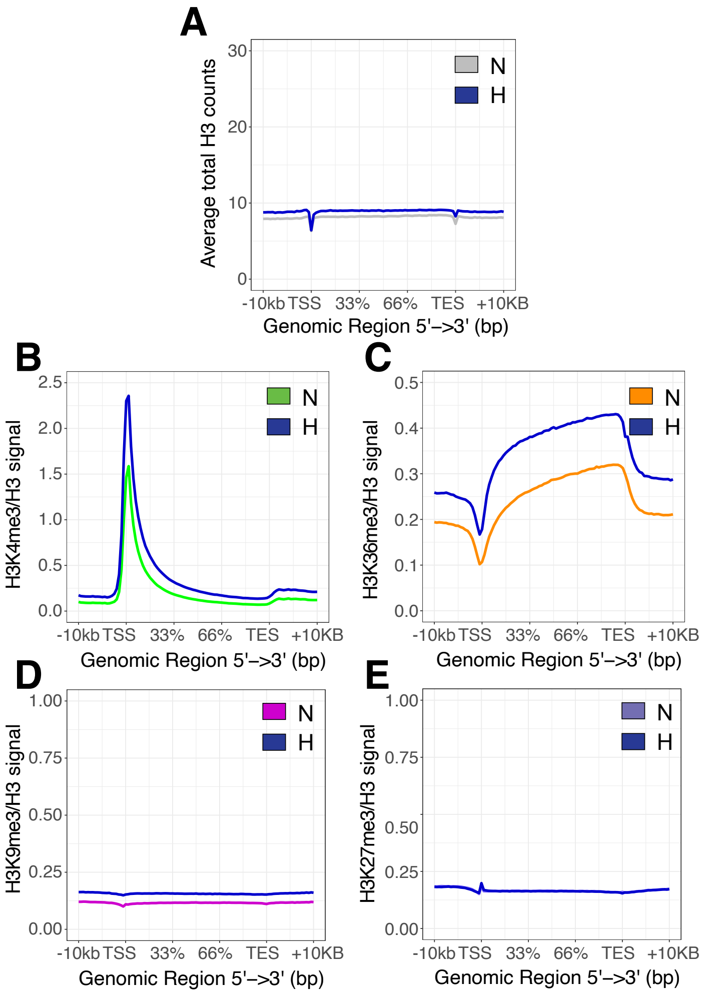

Supplementary figure 3. Hypoxia induces global changes in histone tri-methylation when normalised to histone occupancy. (A) Average profile of total histone ChIP-seq signal in PC3 cells incubated in 21% O2 (grey) or 0.5% O2 (blue) for 16 hours. **(B-E)** Average profiles of H3K4me3 and H3K36me3, H3K9me3 and H3K27me3 ChIP-seq signal normalised to total H3 ChIP-seq signal in PC3 cells incubated in 21% O2 normoxia or 0.5% O2 hypoxia (blue) for 16 hours. Profiles were normalised to Drosophila spike-in control and then to total H3 signal, averaged between n=2 replicates, and plotted across all gene bodies ±10kb.

Supplementary figure 4

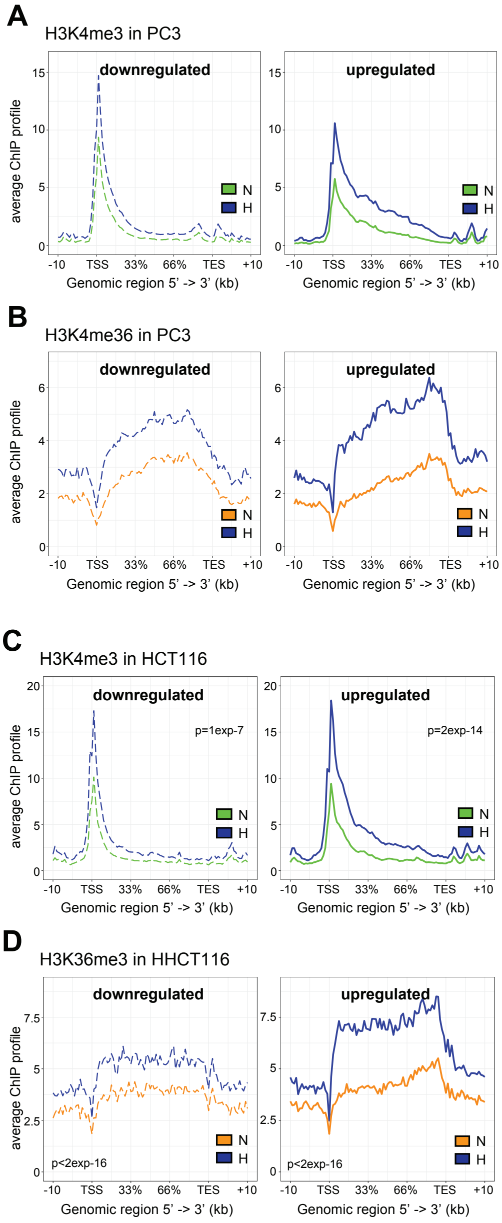

Supplementary figure 4. Hypoxia induces H3K4me3 and H3K36me3 at both up- and downregulated genes. Average ChIP-seq signal, normalised to Drosophila spike-in, for (A) H3K4me3 and (B) H3K36me3 in PC3 cells incubated in 21% O2 normoxia or 16 hours of 0.5% O2 hypoxia (blue) across the 100 most significantly downregulated and 100 most significantly upregulated gene loci, based on the fold-change in mRNA expression from normoxia to hypoxia. (C & D) the same plots for HCT116 cells.

Supplementary figure 5

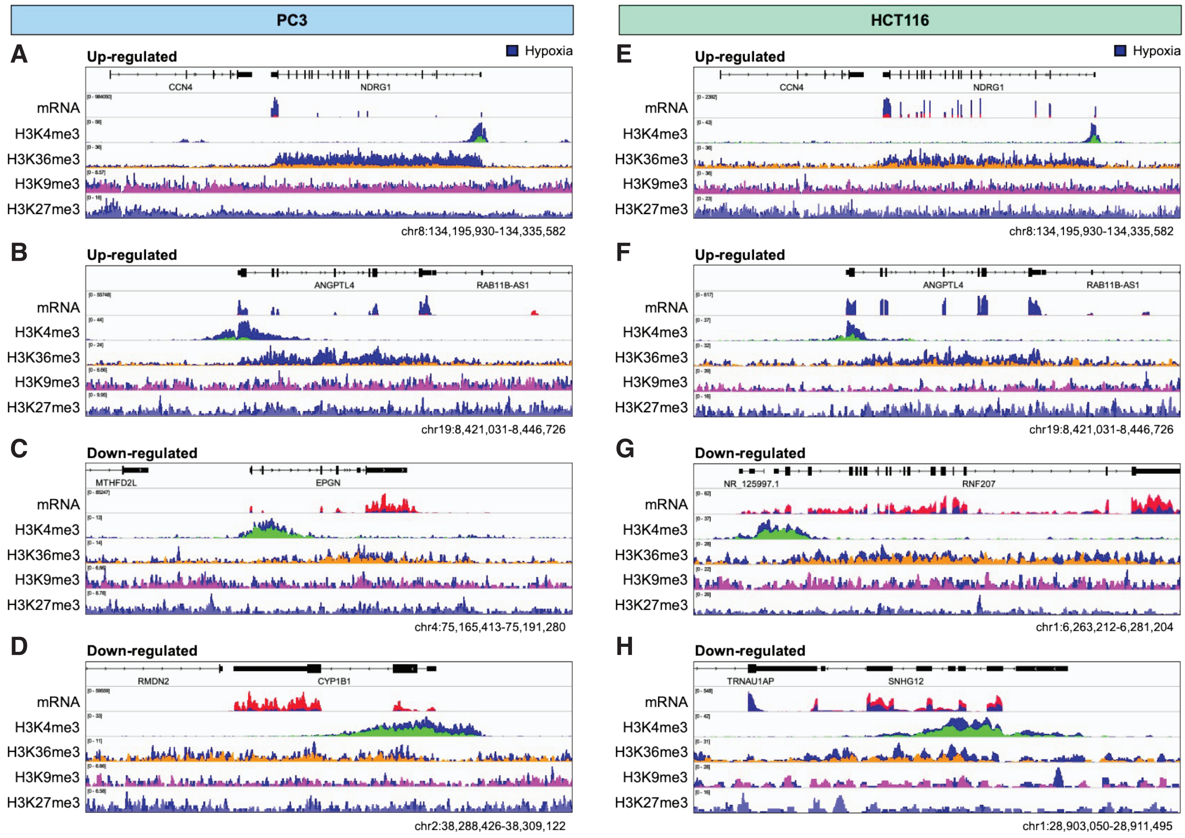

Supplementary figure 5. Hypoxic regulation of histone trimethylation at representative up- and downregulated genes. IGV visualisation of normalised RNA-seq and ChIP-seq signal at the upregulated genes **(A)** *NDRG1* and **(B)** *ANGPTL4*, and downregulated genes **(C)** *EPGN*, and **(D)** *CYP1B1* in PC3 cells and at the upregulated genes **(E)** *NDRG1* **(F)** *ANGPTL4*, and the downregulated genes **(G)** *RNF207* and **(H)** *SNHG12* in HCT116 cells. Each track displays one representative replicate of cells exposed to normoxia (21% O_2) or hypoxia (0.5% O_2 for 16 hours). Normoxia samples for RNA-seq (red), H3K4me3 (green), H3K36me3 (orange), H3K9me3 (maroon) and H3K27me3 (lilac) are shown together with each paired hypoxia sample shown in blue.

Supplementary figure 6

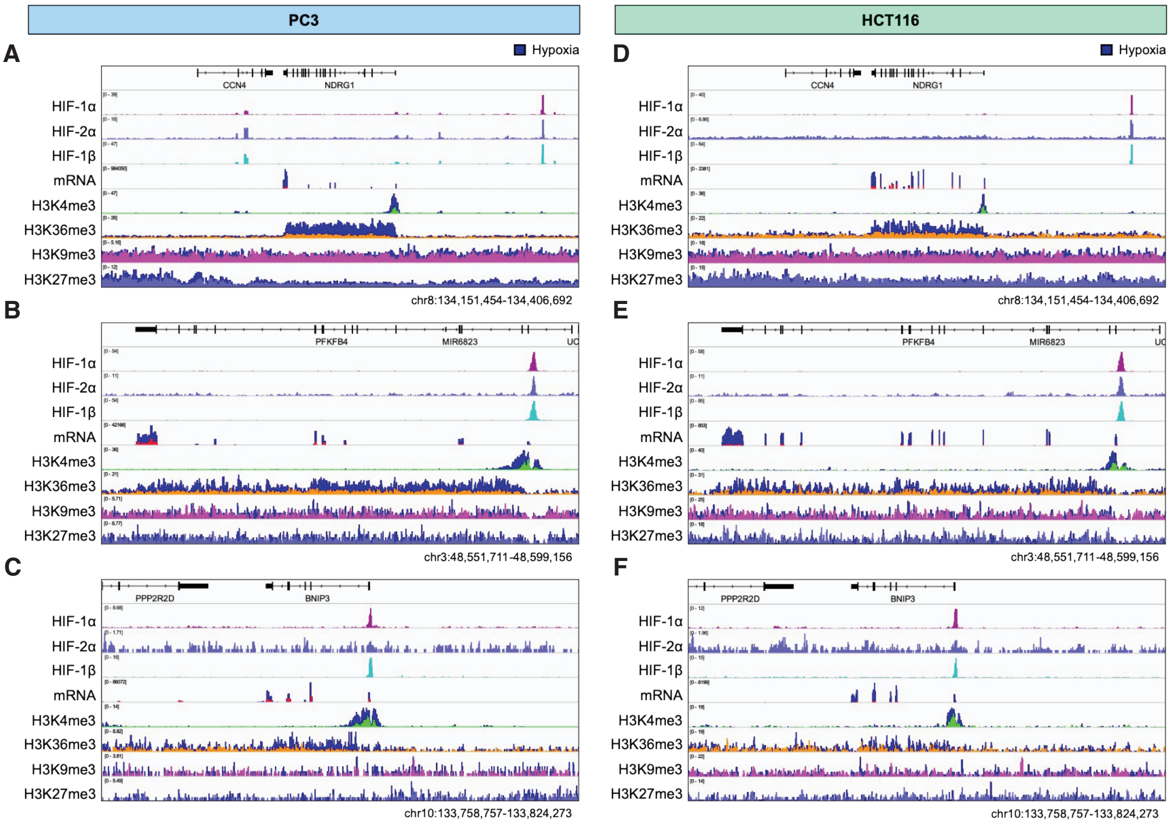

Supplementary figure 6. Hypoxic regulation of histone trimethylation at representative HIF target genes. IGV visualisation of normalised ChIP-seq and RNA-seq signal at the HIF target genes genes **(A)** *NDRG1*, **(B)** *PFKFB4* and **(C)** *BNIP3* in PC3 cells and (D-F) at the same genes in HCT116 cells. Each track displays one representative replicate of cells exposed to normoxia (21% O_2) or hypoxia (0.5% O_2 for 16 hours). HIF-1α, HIF-2α and HIF-1β tracks are shown for hypoxic cells only. Normoxia samples for RNA-seq (red), H3K4me3 (green), H3K36me3 (orange), H3K9me3 (maroon) and H3K27me3 (lilac) are shown together with each paired hypoxia sample shown in blue.

Supplementary figure 7

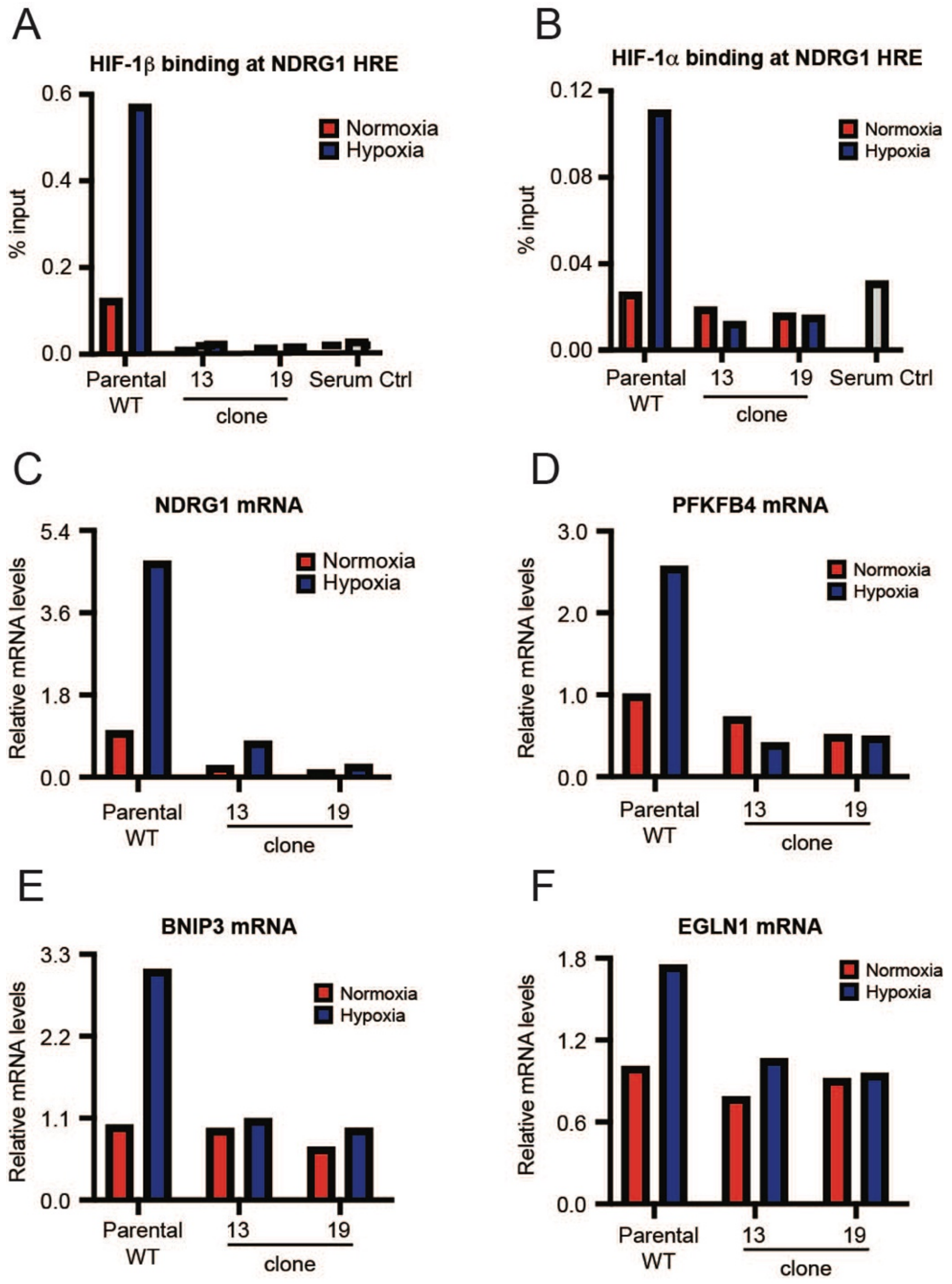

Supplementary figure 7. Attenuation of HIF binding and HIF-target gene induction in HIF-1β knock-out (KO) PC3 cells. ChIP-qPCR analysis of (A) HIF-1β and (B) HIF-1α chromatin binding in parental wild-type (WT) PC3 cells, and two independent HIF-1β KO clones (n=1). RT-qPCR analysis of mRNA levels of the HIF target genes (C) *NDRG1*, (D) *PFKFB4*, (E) *BNIP3*, and (F) *EGLN1* in parental wild-type (WT) PC3 cells, and two independent HIF-1β KO clones (n=1). Cells were incubated in normoxia (21% O_2, red) or hypoxia (0.5% O_2 for 16 hours, blue).

Supplementary figure 8

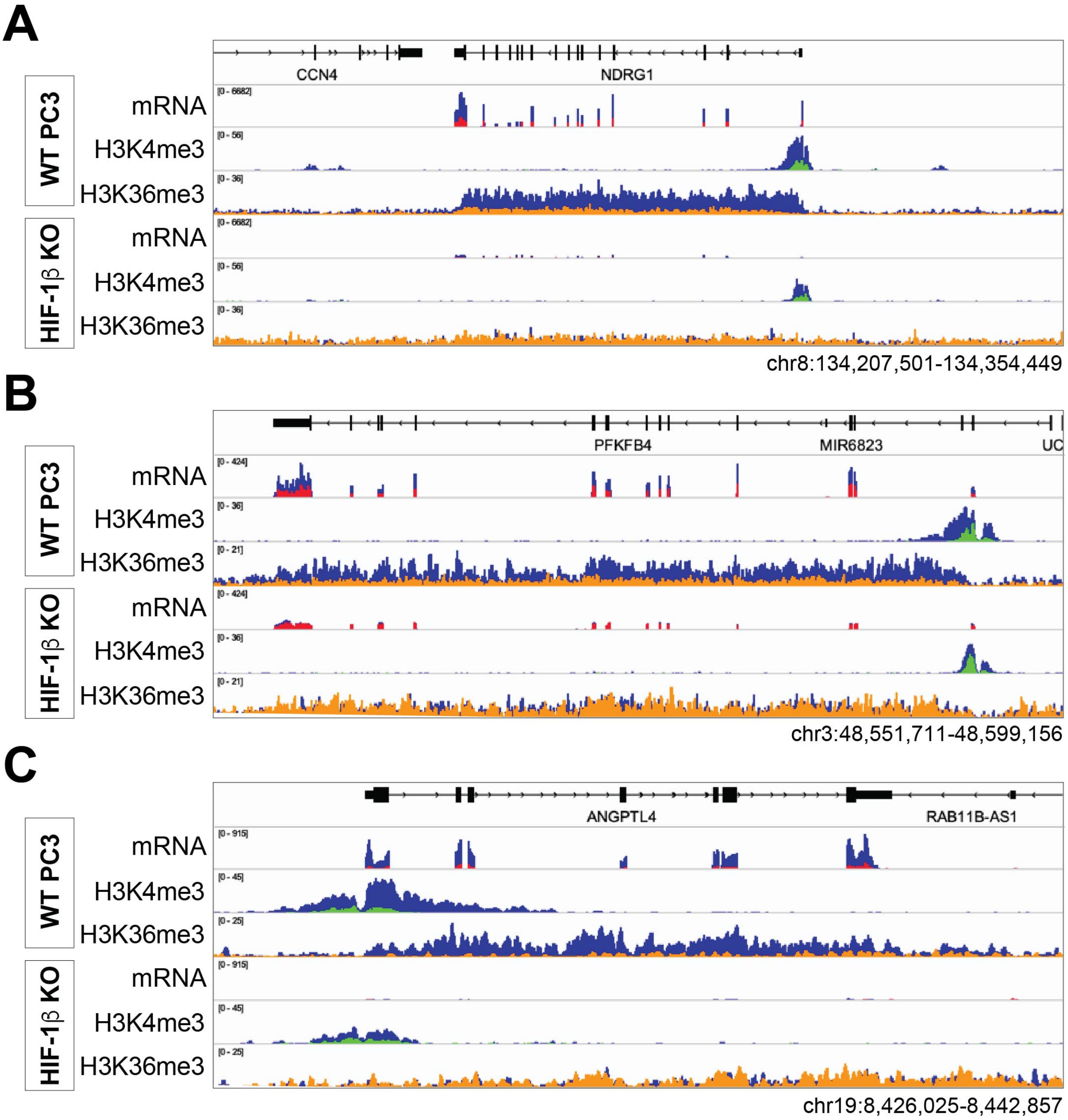

Supplementary figure 8. Hypoxic changes in mRNA and histone trimethylation at selected gene loci. IGV visualisation tracks showing RNA-seq and ChIP-seq signal at the (A) *NDRG1*, (B) *PFKFB4*, and (C) *ANGPTL4* gene loci. Each track displays one representative replicate of parental wild-type (WT) PC3 or HIF-1β KO cells exposed to normoxia (21% O2) or hypoxia (0.5% O2 for 16 hours). Normoxia samples (red, green, orange) overlay each paired hypoxia sample in blue to display site-specific changes in mRNA, H3K4me3, and H3K36me3 at the HIF target genes.

Supplementary figure 9

Figure 1A
Raw Images

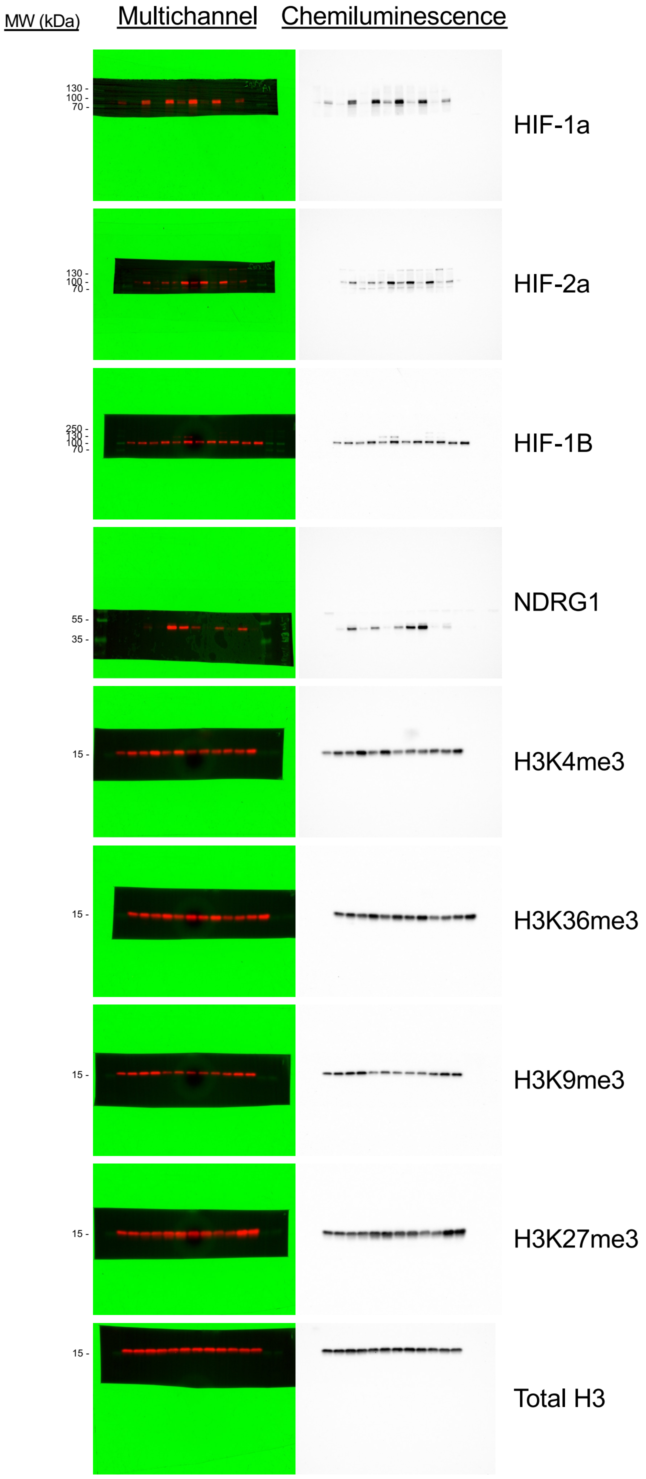

Figure 7A
Raw Images

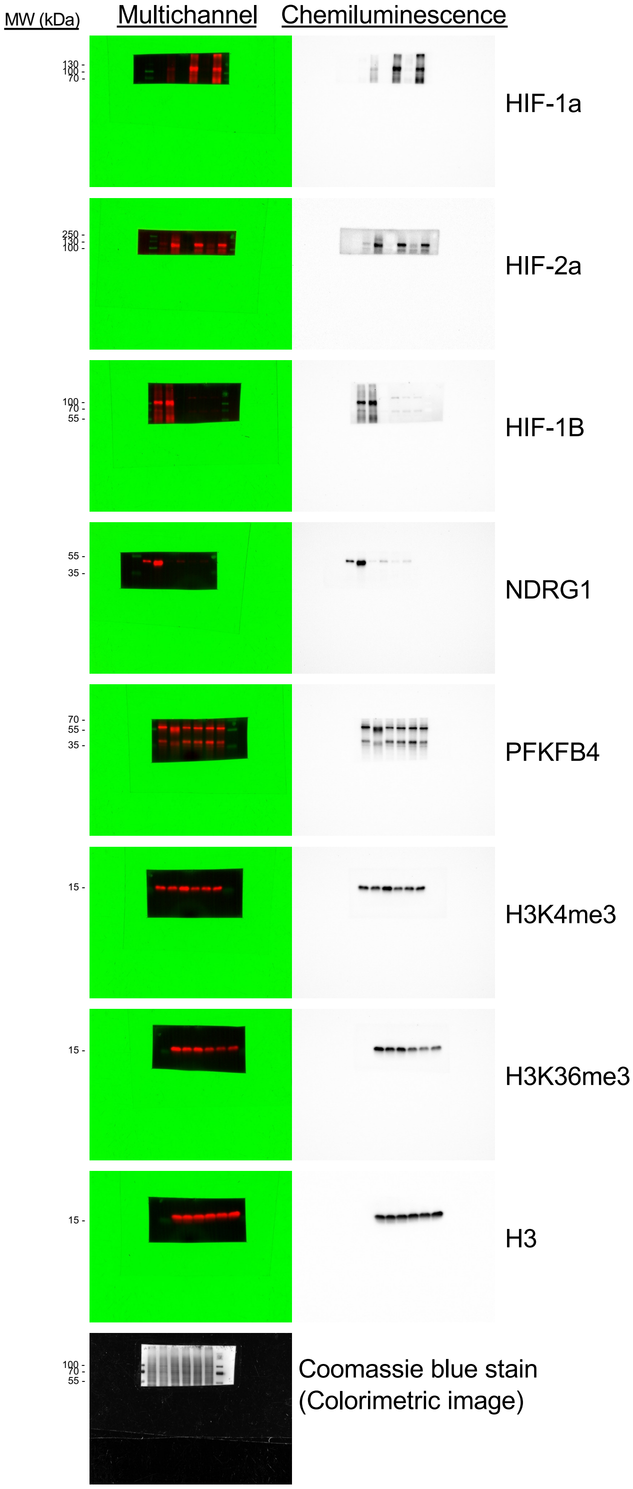

Supplementary figure 9. Raw images for Western blots.
