# Supplementary material for: Hypoxic regulation of chromatin and gene transcription
Source: Commun Biol. 2026 Mar 25;9:665. doi: 10.1038/s42003-026-09875-6 (PMC13181004; doi:10.1038/s42003-026-09875-6)
Supplement: Supplementary file 3 — Description of Additional Supplementary Files [file 42003_2026_9875_MOESM3_ESM.docx]

**Description of Additional Supplementary File**

File name: Supplementary data
Description: The source data and original Western blot images have been submitted as Supplementary data.
